# Impact on continuity of care of decentralized versus partly centralized mental health care in Northern Norway

**DOI:** 10.5334/ijic.674

**Published:** 2011-12-14

**Authors:** Lars Henrik Myklebust, Reidun Olstad, Svein Bjorbekkmo, Martin Eisemann, Rolf Wynn, Knut Sørgaard

**Affiliations:** Psychiatric Research Centre of North Norway, Nordland Hospital Trust, N-8092 Bodø, Norway; Psychiatric Research Centre of North Norway, University Hospital of North Norway, N-9291 Tromsø, Norway; Nordland Hospital Trust, N-8092 Bodø, Norway; Department of Psychology, Faculty of Health Sciences, University of Tromsø, N-9038 Tromsø, Norway; Department of Clinical Medicine, Faculty of Health Sciences, University of Tromsø, UNN-Åsgård, Building 5, N-9291 Tromsø, Norway; Psychiatric Research Centre of North Norway, Nordland Hospital Trust, N-8092 Bodø, Norway

**Keywords:** decentralization, integration, psychiatry, service models, hospitalization, outpatients

## Abstract

**Background:**

The issue of continuity of care is central in contemporary psychiatric services research. In Norway, inpatient admissions are mainly to take place locally, in a system of small bed-units that represent an alternative to traditional central psychiatric hospitals. This type of organization may be advantageous for accessibility and cooperation, but has been given little scientific attention.

**Aims:**

To study whether inpatients’ utilization of outpatient services differ between an area with a decentralized care model in comparison to an adjacent area with a partly centralized model.

**Method:**

The study was based on data from a one-year registered prevalence sample, drawing on routinely sampled data supplemented with data from medical records. Service-utilization for 247 inpatients was analyzed. The results were controlled for diagnosis, demographic variables, type of service system, localization of inpatient admissions, and length of hospitalization.

**Results:**

Most inpatients in the area with the decentralized care model also utilized outpatient consultations, whereas a considerable number of inpatients in the area with a partly centralized model did not enter outpatient care at all. Type of service system, localization of inpatient admission, and length of hospitalization predicted inpatients’ utilization of outpatient consultations. The results are discussed in the light of systems integration, particularly management-arrangements and clinical bridging over the transitional phase from inpatient to outpatient care.

**Conclusion:**

Inpatients’ utilization of outpatient services differed between an area with a decentralized care model in comparison to an adjacent area with a partly centralized care model. In the areas studied, extensive decentralization of the psychiatric services positively affected coordination of inpatient and outpatient services for people with severe psychiatric disorders. Small, local-bed units may therefore represent a favourable alternative to traditional central psychiatric hospitals.

## Introduction

### District psychiatric centres in Norway

Recent research advocates complex mental health service systems that include both community services and psychiatric hospitals [1]. Consequently, the continuity of care issue prevails in contemporary psychiatric services research [2, 3].

The Norwegian Parliament initiated in 1997 a plan on the development of the mental health care system, based upon the overarching objective of strengthening accessibility and continuity of care [4]. Norwegian psychiatric services are now characterized by an extensive decentralization of outpatient services and psychiatric beds, in addition to some remaining central psychiatric hospitals [5, 6]. The present system is divided into three administrative levels:

- The 1st level of municipality services, staffed by semi-specialized personnel and general practitioners.
- The 2nd level of local district psychiatric centres, staffed with psychiatrists, clinical psychologists, and psychiatric nurses.
- The 3rd level of central psychiatric hospitals, also staffed with psychiatrists, clinical psychologists and psychiatric nurses.

According to the national guidelines [5, 6], the district psychiatric centres are the central element in this system because of their responsibility for providing specialized psychiatric care locally, as well as coordinating all the other mental health services of a sector. The local municipality services are staffed with semi-specialized personnel under the authority of the council physician, and served by the local general practitioners [5]. They provide outpatient counselling services as well as long-term residential care for severely mentally ill persons, in the form of sheltered homes [6].

Although this organization may be advantageous for local accessibility, integration and cooperation between the different components has received relatively little scientific attention so far [7–11]. The theme is highlighted in this ecological study of the services in the County of Nordland in North Norway.

### Continuity of care

There is little knowledge about the outcomes of systems organization on patients’ health. The dynamic relationship between organizational aspects and individual characteristics of patients has been difficult to establish probably because the pathway between organizational variables and client change is mediated by a complex webbing of factors [12, 13]. The concept of continuity of care has therefore been suggested as a more testable proxy for clinical outcome [3]. It represents the uninterrupted treatment for individuals suffering from serious psychiatric disorders, and has long been a key concept in the evaluation of deinstitutionalized psychiatric care systems [4, 14–16]. We decided to use findings associated with the concept of continuity of care in the analysis.

Supporters of the concept of continuity of care point to the benefits of combined treatment [16], while critics emphasize the disruptiveness that may occur in recurrent transitions of patients [17] or even the fostering of dependency and a chronic sick role [18]. Despite these controversies, recent results suggest a positive relationship between continuity of care and important health outcomes for severely ill patients, such as quality of life, better community functioning, lower severity of symptoms, and greater service satisfaction [19, 20].

Research on continuity of care has been limited by inconsistent definitions and operational measures [12], and the concept is even regarded as over-used due to its face validity and immediate appeal [18]. Although research lately has concentrated on the development of multi-faceted instruments [21], one frequently used definition of the construct entails a cross-sectional aspect that denotes the cooperation and accessibility of services [12]. Related to this, continuity of care for individual patients may be affected by the degree of systems integration, and in particular by transitions between inpatient and outpatient modes of treatment [3, 20, 22].

### Transitions

Effective clinical bridging seems important to avoid gaps in the delivery of services [23]. The Transitional Discharge Model elaborates this further to emphasize patients’ continuous contact with an entrusted health professional to facilitate the discharge from the psychiatric hospital to the community [24].

In one of the two study areas (see Study areas section for a closer description), the same clinical staff provide both inpatient and outpatient services. Consequently, most patients here have one main therapist throughout their treatment. In the other study area, however, the opportunity to maintain contact between the therapist and the patient across these service modalities is limited, due to a large geographical distance between the district psychiatric centre and central psychiatric hospital. The majority of patients must therefore relate to at least two different therapists if they are to utilize both modes of treatment. Consequently, with respect to the opportunity to maintain a therapeutic alliance throughout treatment, the decentralized care system could be advantageous to the partly centralized system.

### Systems integration

The concept of ‘systems integration’ includes constructs, such as ‘cooperation’, ‘coordination’ and ‘collaboration’, but the terminology has no agreed upon usage. Systems integration may be considered as vertical or horizontal, depending on whether it takes place at various levels of government, among services, on the basis of geography (e.g. sector) or specialization [16]. In our study, the vertical integration perspective is possibly most relevant, due to the organization of Norwegian psychiatric services on the basis of geographically defined sectors.

A key-element in systems integration is managerial arrangements [3]. Voluntary relations between autonomous agencies often reflect low levels of integration, in contrast to the higher levels of integration promoted by overarching units of authority [20]. Clinical administration of most inpatients in the decentralized care system are managed by the head psychiatrist at the local district psychiatric centre, while in the partly centralized system this is done by joint arrangement of the staff at the local district psychiatric centre and the central psychiatric hospital. Due to these differences in management, the decentralized care system could be advantageous to the partly centralized system with respect to the continuity of care for patients with severe illness.

### Aims

We wanted to study the effect of the structure of the psychiatric services on utilization, and whether a decentralized care model was associated with better integrated services and more cooperation for patients with severe psychiatric disorders than a partly centralized care model. Two specific scientific research questions were addressed:

1. Does inpatients’ utilization of outpatient services differ between an area with a decentralized care model in comparison to an adjacent area with a partly centralized model?
2. Are system variables or patient variables the most important in predicting patterns of outpatient services utilization for these patients?

## Methods

### Study areas

The neighbouring study areas comprising the district psychiatric centres of Vesterålen and of Lofoten are of scientific interest because of striking organizational dissimilarities, while at the same time having a strong resemblance in the characteristics of the catchment areas [25].

**Figure 1.**
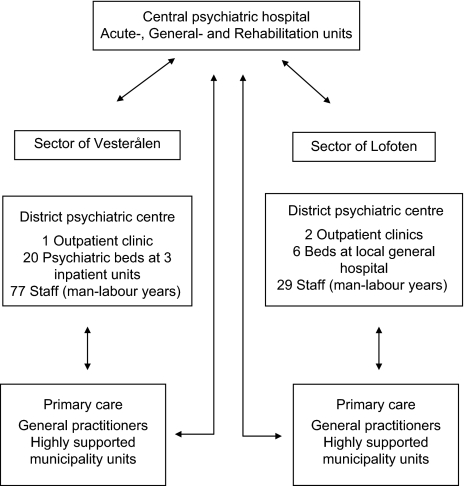
Outline of the psychiatric services in the two sectors of Vesterålen and Lofoten, County of Nordland, Norway.

The main organizational difference lies in the location of psychiatric beds. In Vesterålen, 70% of total inpatient stays are at the local district psychiatric centre, whereas the remaining 30% are at the central psychiatric hospital in Bodø. In Lofoten, this is reversed, with 90% of the inpatient stays at the hospital in Bodø, and only 10% are admitted locally (i.e. in the local somatic hospital) [26]. Consequently, the two systems may be termed a ‘decentralized care model’ (i.e. in Vesterålen) as opposed to a ‘partly centralized care model’ (i.e. in Lofoten). See Figure 1.

The considerably larger staffing at the district psychiatric centre of Vesterålen is mainly due to the requirements of the psychiatric inpatient units. Of the total number of staff employed, 57 are ward staff. By dividing the 20 clinicians (psychiatrists, clinical psychologists and specialist nurses) working at the centre, there are 1.1 outpatient clinicians per 1000 inhabitants. At Lofoten district psychiatric centre, all local psychiatric clinicians work at the two outpatient units. When needed, beds are available for psychiatric patients at the local general hospital in Lofoten, and these patients are cared for by staff otherwise working at somatic units in collaboration with psychiatric staff working at the outpatient units. Consequently, this gives a higher rate of 2.0 outpatient clinicians per 1000 inhabitants. The central psychiatric hospital does not provide outpatient services to patients from the local catchment areas in question. The similarities of the catchment areas’ characteristics are further illustrated in Table 1.

### Design and analyses

The present study was an ecological case study of psychiatric inpatient and outpatient services in two areas, with a secondary analysis of health databases. The design was based upon the routine case-registries of the two district psychiatric centres and the central psychiatric hospital. The records/registries were linked by the patients’ 11-digit social identity numbers. Patients were assigned to the catchment-areas based on their registered home addresses. Missing data were collected from medical records. Data relating to other types of mental health services (including primary care) were unavailable to us.

The study was approved by all relevant agencies, including the Regional Medical Ethics Committee, the Norwegian Data Protection Agency, and The Norwegian Directorate for Health.

The sample was a one-year registered prevalence sample. Patients treated during the year 2005 were included in the study. The sample was limited to patients between 18 and 65 years. A total of 1865 single treatment episodes were aggregated on 1253 patients.

The clinical diagnoses were grouped into four broad categories based on ICD-10 diagnostic criteria: substance abuse disorders (F10–19), psychotic disorders (F20–29), affective disorders (F30–39), and anxiety disorders (F40–49). These accounted for 80.4% of the total sample. Low-frequent diagnoses and psychiatric observations were grouped into the category of ‘other’ (19.6%).

We extracted 247 patients with at least one inpatient stay during the observational period and calculated total utilization of services (i.e. sum of all inpatient stays in total number of days and total number of outpatient consultations), with no temporal differentiation between pre- and post-discharge patterns. Differences in patients-ratio were tested by standard χ^2^. Differences in utilization were tested by Mann-Whitney U-test, due to a skewed distribution.

To control for possible confounders, we performed a logistic regression modelling with the dependent variable ‘In outpatient care (y/n)’. The covariates of service-system and bed-location (local, central) were entered in order to disentangle organizational aspects other than the sheer location of beds. The variables of diagnosis (substance abuse disorders, psychotic disorders, affective disorders, and anxiety disorders) and demographics (gender and age) were entered to control for possible differences in inpatient group characteristics. Due to earlier findings in the field [19, 22], the covariate length of inpatient stay was also entered. In accordance with regressions assumptions, this variable was log-transformed because of a highly skewed distribution.

## Results

The results are presented in Tables 2 and 3. Table 2 shows differences in inpatients’ length of stay between the study areas. The median inpatient stay was twice as long in Vesterålen (i.e. with a decentralized care model) compared to Lofoten (i.e. with a partly centralized care model) (Z=–2.078, p=0.38). Further, most inpatients in the decentralized model also utilized outpatient consultations, whereas a considerable number of inpatients in the partly centralized model did not enter outpatient care at all (Pearson χ^2^=26.522, d.f.=1). A ϕ -coefficient of 0.326 (p=0.001) indicated a medium effect-size of service model [28]. Inpatients in the decentralized care model also had more outpatient consultations than inpatients in the partly centralized model (Z=–2.303, p=0.021).

Table 3 displays the logistic regression model, predicting whether the inpatients had utilized outpatient consultations or not. The total model containing all predictors was statistically significant (χ^2^=51.764, d.f.=10, p<0.000), with an overall goodness of fit at 256.321 (p<0.000). A sensitivity of 46.2% and specificity of 87.0% indicated that it could distinguish between responders with or without outpatient treatment. The model explained between 18.9% (Cox and Snell R Square) and 26.5% (Nagelkerke R Square) of the total variance.

The only three covariates with a unique and statistically significant contribution were service-system, central admission only and length of inpatient stay. Service-system emerged as the strongest predictor, indicating that an inpatient in the decentralized care system was more than three times more likely to receive outpatient care compared to inpatients in the partly centralized system. Further, if an inpatient in either system was admitted to the central hospital only, it negatively predicted utilization of outpatient consultations at the local level. Longer inpatient stays increased the likelihood of also utilizing outpatient consultations.

## Discussion

### General considerations

The study showed that most inpatients in the decentralized system also utilized outpatient consultations, whereas a considerable number of inpatients in the partly centralized system did not enter outpatient care at all, despite a higher ratio of clinical outpatient staff being available in Lofoten. The type of service-system, the localization of the inpatient admissions, and the length of the inpatient stays predicted inpatients’ utilization of outpatient consultations.

Differences in systems integration could explain these results [3]. In the partly centralized care system, clinical management of inpatient and outpatient modes of treatment were characterized by collaborative efforts between highly autonomous clinical units at the central psychiatric hospital and district psychiatric centre. In the decentralized care system on the other hand, the local management at the district psychiatric centre was in charge of both inpatient and outpatient treatments for most patients. A split management may in several ways hamper the coordination of services for individual patients.

One explanation of why the coordination of services between the central hospital and the local service providers is difficult is the limited availability of hospital beds, which may lead to an early discharge of patients before services have been fully coordinated [18, 29]. Moreover, the coordination of services may fail because of the providers’ guard of their organizational borders and struggle for control [30]. There may also be divergent clinical opinions and ideological cultures between a local community service and a traditional psychiatric hospital [31]. A related aspect may be the possibility for exchange of clinical information, which has been found to be of importance to prevent drop out from care after discharge [32]. Strict rules on patient confidentiality or staff located at different institutions may hamper effective use of clinical information between the district psychiatric centre and the central psychiatric hospital in the partly centralized care system. In the decentralized care system, on the other hand, shared medical records and overlapping staff for both inpatient and outpatient treatment may facilitate the flow of clinical information.

The opportunity for therapeutic continuity between individual patients and therapists may also explain the differences between the two systems [24]. Complex systems of services may overburden the ability of severely ill patients to orientate and adapt to several settings [17]. In particular, the prevention of non-adherence after hospitalization requires tailored interventions, where an appointment with a clinician is central [33]. Certainly, this may be easier to establish in a decentralized care system. The opportunity to maintain a trusted relationship over the transitional phase may be reduced in the partly centralized care system because of the considerable geographical distance between the central psychiatric hospital and the district psychiatric centre. Patients must therefore often establish contact with different therapists depending on whether they are in inpatient or outpatient care. In the decentralized care system on the other hand, the same clinician is usually responsible for individual patients in both modes of treatment.

An alternative interpretation could be that clinicians in the partly centralized care system regard the best outpatient treatment for severely ill to be in primary care. If this were the case, it could potentially be problematic as severely ill psychiatric patients usually need coordinated services between specialists and general practitioners [34–36]. However, our data do not allow for a further analysis of whether the two systems differ in the degree of outpatient treatment in primary care.

The present study serves as a good case illustrating several important concepts in the integrated care literature, including autonomy versus integration, vertical integration, and continuity of care [37–39].

With respect to the theme of autonomy versus integration of services; in our case, the partly centralized care system represents autonomous clinical units at a central and a district level, respectively. The decentralized care system, on the other hand, represents local services, where the services are highly coordinated and overlapping.

Moreover, in the partly centralized care system, the psychiatric hospital beds are located on a tertiary level of care and the outpatient services on a secondary level of care. Accordingly, the case is an illustration of services (i.e. in the partly centralized care system) where vertical integration has not been accomplished.

Furthermore, in our case, we have discussed how the partly centralized system affects continuity of care negatively. One aspect is the lack of shared information, as medical records and other relevant information is not as effectively shared between providers at the central psychiatric hospital and at the district psychiatric centres. Another aspect is the early discharge and lack of coordinated planning of discharge from the central psychiatric hospital. In addition, there is the topic of lack of therapist continuity, where patients in the partly centralized care system must relate to different therapists, depending on whether they are treated in the central psychiatric hospital or in the district psychiatric centre. However, other aspects of the services are also of importance to continuity of care and integration of services (such as service delivery processes and quality of care), these are not dealt with in the present study.

### Strengths and limitations

The study has several strengths. The close to natural experimental design of the study represents a major advantage. The geographical delimitations of the catchment areas, the similarity in socio-demography [25], and the absence of private health-service providers render a good opportunity to control for confounders. The possibility to reveal individual patient’s total utilization-patterns over independent specialist services is also a strength of the study.

The importance of ecological studies in a range of different areas for informed policy planning has previously been highlighted in several studies [9–11, 40]. This approach may be especially applicable in countries where integrated inpatient and outpatient care is in place, such as parts of France [7] and Norway [26, 40].

While we may not generalize on the basis on the results of our study of psychiatric services in two North-Norwegian areas, we believe the present study may provide a basis for future studies in this field in Norway as well as in other countries.

The relatively small sample size may compromise the ability to generalize the results. However, the fact that significant effects were found indicates strong associations. A related aspect is the restricted number of variables on patients’ characteristics used in the regression model. A closer assessment of individual clinical and psychosocial problems could reduce the importance of system variables.

Our design does not adhere to a very restricted definition of ‘continuity of care’ i.e. the follow-up after discharge from hospital. We nevertheless consider parts of the literature on this concept as relevant in the discussion of our results.

Our model explained between 18.9% and 26.5% of the variation in use of outpatient services, and other factors were therefore also likely to be of importance. Data relating to other types of mental health services (including primary care and long-term residential care) were unavailable to us and we cannot therefore further evaluate the possible impact of these factors on the utilization of outpatient services. We lack data on several other aspects of importance to the integration of the psychiatric services, including data on service delivery processes, case management, IT-services, and data on the quality of care, and are therefore unable to address these topics.

Also, our design has limitations inherent in the defined observational period. The censorship issue concerning patients hospitalized before the start of the period, or not yet discharged by the end of the period, could possibly affect the conclusions. This is particularly relevant as the sample size of inpatients is low and outliers or late inception cases may have a significant impact in any of the two areas. Probably, the analysis of a series of years could have provided more robust findings. To some degree, this has been controlled for by including length of inpatient stay as a covariate in the regression model. A more thorough survival analysis was not possible based on the present data. Modeling techniques, such as Bootstrap and Monte Carlo could have helped to overcome the problem of a low sample size of inpatients in both areas and the missing data due to the use of a calendar year, and should be considered for future studies.

Future studies could benefit from a different design, for instance by including a longer observational period and thereby a higher number of complete courses of treatment or alternatively, by analyzing a fixed time period for each patient. It would also be interesting to compare data before and after the introduction of local inpatient care.

## Conclusions

Inpatients’ utilization of outpatient services differed between an area with a decentralized care model in comparison to an adjacent area with partly centralized services. In the areas studied, extensive decentralization of the psychiatric services positively affected coordination and integration of inpatient and outpatient services for people with severe psychiatric disorders. Small, local-bed units may therefore represent a favourable alternative to traditional central psychiatric hospitals.

## Figures and Tables

**Table 1.** Characteristics of catchment areas for the sectors of Vesterålen and of Lofoten, County of Nordland, Norway

Variable		Sector of Vesterålen		Sector of Lofoten	
Geography	Distance to central psychiatric hospital over land (km)	329.6		457.2	
	Travel time by air (min)	30		25	
	Size (square km)	2510.6		1197.3	
Infrastructure	Municipalities	5		4	
	Cities	2		2	
	Airports	2		2	
	Larger harbours	2		2	
Catchment area demographics	Persons aged 18–65	18,212		13,417	
	Gender	Male	Female	Male	Female
	Young^2^	2082 (11.4%)	1899 (10.4%)	1641 (12.2%)	1474 (11.0%)
	Middle aged	4111 (22.6%)	3989 (22.0%)	3029 (22.6%)	2916 (21.7%)
	Elderly	3147 (17.3%)	2984 (16.4%)	2285 (17.0%)	2072 (15.4%)
	Sum	9340 (51.3%)	8872 (48.7%)	6955 (51.8%)	6462 (48.2%)
	Population density (persons per square km)	12.1		18.7

^1^Year of 2005 by Statistics Norway [27]. ^2^Young: 18–29, middle aged: 30–49, elderly: 50–65.

**Table 2.** Utilization pattern for patients in a decentralized care versus a partly centralized care model of psychiatric services. One-year registered prevalence sample (2005)

Study area/Service-system	Lofoten/Partly centralized care model		Vesterålen/Decentralized care model	
Category of patients	Total patient population	Patients with at least one inpatient stay	Total patient population	Patients with at least one inpatient stay
Number of patients	546	106	716	141
Gender					
Male	231	47	284	64
Female	315	59	432	77
Age (mean)	38.2 (sd=11.9)	41.0 (sd=12.5)	38.6 (sd=12.2)	40.7 (sd=13.3)
Length of inpatient stays (in days, median)	…	10*	…	20*
Outpatient consultations (median)	4*	5*	3*	7*
Patients (n) receiving outpatient consultations	484 (88.2%)**	54 (50.9%)**	679 (94.3%)**	115 (81.6%)**

*p<0.05, **p<0.01, when service-models were compared.

**Table 3.** Logistic regression model of inpatients’ utilization of outpatient care

Variable	B	Sig.	OR	95% CI
Gender	0.236	0.474	1.266	0.663–2.417
Age	–0.009	0.470	0.991	0.967–1.016
Substance abuse disorders	–0.342	0.566	0.711	0.221–2.282
Psychotic disorders	–0.444	0.449	0.641	0.203–2.027
Affective disorders	0.336	0.551	1.399	0.463–4.227
Anxiety disorders	–0.479	0.381	0.619	0.212–1.808
Length of inpatient stay	0.827	0.003	2.286	1.323–3.951
Type of service system	1.120	0.001	3.065	1.555–6.044
Local admission only	–0.310	0.626	0.733	0.210–2.555
Central admission only	–1.526	0.011	0.217	0.067–0.705
Constant	0.315	0.721	1.370	
